# Association of Triglyceride–Glucose Index with Angiographic Thrombus Burden in Patients with ST-Elevation Myocardial Infarction: A Prospective Observational Study

**DOI:** 10.3390/jcm15124793

**Published:** 2026-06-20

**Authors:** Nikolaos Stalikas, Marios G. Bantidos, Efstratios Karagiannidis, Athina Nasoufidou, Sara Corradetti, Anthony Kechichian, Christos Kofos, Maria Fasoula, Matthaios Didagelos, Marios Sagris, Barbara Fyntanidou, Antonios Ziakas, Theodoros Karamitsos, Georgios Giannopoulos

**Affiliations:** 1 Cardiovascular Center Aalst, AZORG Ziekenhuis, Moorselbaan 164, 9300 Aalst, Belgium; sara.corradetti@uniroma1.it (S.C.); anthony.kechichian@gmail.com (A.K.); 2 Department of Emergency Medicine, AHEPA General University Hospital of Thessaloniki, Aristotle University of Thessaloniki, 54636 Thessaloniki, Greece; stratoskarag@gmail.com (E.K.); m.occludent@gmail.com (M.F.); bfyntan@yahoo.com (B.F.); 3 Second Department of Cardiology, Hippokration General Hospital of Thessaloniki, Aristotle University of Thessaloniki, 54642 Thessaloniki, Greece; athinanassi@gmail.com (A.N.); chriskofos21@gmail.com (C.K.); 4 Department of Clinical and Molecular Medicine, Sapienza University of Rome, 00185 Rome, Italy; 5 First Department of Cardiology, AHEPA General University Hospital of Thessaloniki, Aristotle University of Thessaloniki, 54124 Thessaloniki, Greece; manthosdid@yahoo.gr (M.D.); aziakas@auth.gr (A.Z.); tkaramitsos@auth.gr (T.K.); 6 Department of Cardiology, Tzaneio General Hospital of Piraeus, 18536 Piraeus, Greece; masagris1919@gmail.com; 7 Third Department of Cardiology, Hippokration General Hospital of Thessaloniki, Aristotle University of Thessaloniki, 54642 Thessaloniki, Greece; ggiann@auth.gr

**Keywords:** acute coronary syndrome (ACS), coronary angiography, distal embolization, insulin resistance (IR), ST-elevation myocardial infarction (STEMI), thrombus burden, triglyceride–glucose index (TyG)

## Abstract

**Background:** The triglyceride–glucose (TyG) index has emerged as a simple surrogate marker of insulin resistance and metabolic disruption. In the context of ST-elevation myocardial infarction (STEMI), such disturbances have been associated with adverse cardiovascular outcomes, more complex angiographic profiles, and microvascular complications. However, data on the association between TyG and intracoronary thrombus burden (TB) in STEMI remain limited. **Methods:** In this prospective observational study, we included consecutive STEMI patients treated with primary percutaneous coronary intervention (pPCI). The TyG index was calculated using the following formula: ln [fasting triglycerides (mg/dL) × fasting glucose (mg/dL)/2]. TB was graded according to the modified thrombolysis in myocardial infarction (mTIMI) thrombus classification score after restoration of antegrade flow with a wire or small balloon when the culprit vessel was initially totally occluded. Patients were categorized as low-TB (LTB; mTIMI grades 1–3) and high-TB (HTB; mTIMI grade 4). The primary outcome was HTB; secondary outcomes were distal embolization and no-reflow. Associations between TyG and outcomes were assessed using univariable and multivariable logistic regression, restricted cubic spline analysis, and receiver operating characteristic (ROC) curves to evaluate incremental predictive value. **Results:** A total of 309 patients were analyzed. The TyG index was significantly higher in the HTB group compared with the LTB group (9.12 ± 0.62 vs. 8.92 ± 0.64, *p* = 0.004). In a stepwise multivariable model, TyG remained independently associated with HTB (adjusted odds ratio = 1.61; 95% confidence interval: 1.11–2.37; *p* = 0.014). Adding TyG to a baseline clinical model only numerically improved discrimination for HTB, as reflected by a small increase in ROC area under the curve. Restricted cubic spline analysis demonstrated a monotonic rise in the probability of HTB with higher TyG values. Higher TyG also showed non-significant trends toward increased odds of distal embolization and no-reflow. **Conclusions:** The TyG index was independently associated with HTB in STEMI patients undergoing pPCI and may serve as an accessible adjunctive marker for incremental risk stratification beyond conventional clinical and angiographic factors.

## 1. Introduction

The triglyceride–glucose (TyG) index, calculated from fasting triglyceride and glucose levels, has emerged as a convenient and reproducible surrogate marker of insulin resistance (IR) and broader metabolic derangement [1]. It demonstrates comparable performance and in some cases superior discriminatory ability when compared to established reference methods [2].

IR, the defining feature of metabolic syndrome and type 2 diabetes mellitus, is known to promote both systemic inflammation and a prothrombotic state [3]. This effect is mediated through several mechanisms, including endothelial dysfunction, oxidative stress, platelet hyperreactivity, and elevated circulating procoagulant factors [4,5,6,7]. These mechanisms are not only chronic contributors to atherogenesis but may also acutely exacerbate vascular injury and ischemic events. Thus, an elevated TyG index likely not only reflects metabolic dysfunction but also indicates a heightened inflammatory and thrombotic milieu within the cardiovascular system.

Emerging evidence supports the prognostic value of the TyG index across the cardiovascular disease (CVD) spectrum, with higher TyG levels associated with the presence and greater complexity of coronary artery disease (CAD) [8,9]. Moreover, in acute myocardial infarction (AMI), elevated TyG has been linked to poorer short- and long-term outcomes, including higher mortality and increased rates of major adverse cardiovascular events (MACEs) [2,10,11].

Current understanding of thrombus formation in STEMI increasingly extends beyond a purely mechanical or hemodynamic process [12,13]. Clinical data have shown that systemic inflammation and IR correlates with thrombus burden (TB) and impaired coronary flow [14]. Nevertheless, despite the well-established adverse impact of TB on reperfusion success and procedural outcomes, the ability of the TyG index to prognosticate TB in the acute STEMI setting has been investigated in only a limited number of studies [2,15,16,17,18].

In the present study, we aimed to evaluate whether TyG index is associated with increased TB, as well as with other key procedural outcomes, including distal embolization, no-reflow, final thrombolysis in myocardial infarction (TIMI) flow grade, and residual thrombus, in patients presenting with STEMI.

## 2. Materials and Methods

### 2.1. Study Population

We performed a single-center, prospective observational study conducted between 2018 and 2021 at AHEPA University Hospital, in Thessaloniki, Greece. Consecutive adult patients presenting with STEMI and treated with pPCI were screened for inclusion. The diagnosis of STEMI was based on the criteria of the Fourth Universal Definition of Myocardial Infarction [19]. Patients with non-ST-elevation acute coronary syndromes (NSTE-ACS) [non-ST-elevation MI (NSTEMI) or unstable angina (UA)], prior coronary artery bypass grafting (CABG), or missing data required to calculate the TyG index were excluded.

### 2.2. Data Collection and Definitions

Baseline demographic characteristics, cardiovascular risk factors (e.g., hypertension, diabetes mellitus, dyslipidemia, smoking status, kidney disease), previous history of CAD, and clinical presentation were recorded at admission using standardized case report forms. Laboratory parameters, including serum glucose and triglyceride levels, were obtained from blood samples drawn according to the study’s protocol in patients with fasting of at least 6 h. The TyG index was calculated using the following formula:

$$ln\;[\frac{fasting\;triglycerides\;(mg/dL)\times fasting\;glucose\;(mg/dL)}{2}]$$

TB in the culprit vessel was assessed on coronary angiography using the modified TIMI (mTIMI) thrombus classification score, after restoration of antegrade flow with a guidewire or small balloon in cases of initial total occlusion (TIMI thrombus grade 5) [20,21]. Based on this classification, patients were categorized as low-TB (LTB, grades 1–3) and high-TB (HTB, grade 4).

Distal embolization was defined as the angiographic appearance of a new, abrupt vessel cut-off or intraluminal filling defect in a distal segment or side branch, not present on the initial angiogram, with preserved proximal antegrade flow [22]. No-reflow was defined as inadequate myocardial perfusion through a given coronary segment (final TIMI flow grade < 3) in the absence of angiographic evidence of mechanical vessel obstruction (e.g., dissection, spasm, or significant residual stenosis) [23]. Final TIMI flow grade and residual thrombus grade were recorded at the end of the procedure.

### 2.3. Coronary Angiography and PCI

All patients underwent emergent coronary angiography and pPCI via radial or femoral access, according to operator preference and current practice standards. Culprit lesions were treated with balloon angioplasty and stent implantation as appropriate. The use of manual thrombus aspiration, glycoprotein IIb/IIIa inhibitors, intracoronary vasodilators, and other adjunctive pharmacotherapies was left to the discretion of the interventional cardiologist.

Angiographic analyses, including assessment of thrombus grade, distal embolization, no-reflow, final TIMI flow, and residual thrombus, were performed by experienced interventional cardiologists blinded to TyG index values. Disagreements were resolved by consensus.

### 2.4. Ethical Approval

The study was conducted in accordance with the Declaration of Helsinki [24]. The broader prospective STEMI/thrombus-burden research protocol was approved by the Bioethics and Deontology Committee of the Aristotle University School of Medicine (Protocol No. 373; 5 July 2017) and by the Scientific Board of AHEPA University General Hospital (Protocol No. 38; 25 January 2018), with a planned duration of 48 months. This 48-month approval period covered the reported 2018–2021 recruitment and follow-up period. The related metabolic/stress hyperglycemia continuation was subsequently approved by the Directory Board and the Scientific Council of AHEPA University General Hospital (Protocol No. 321/2019; 6 February 2019). All study-related procedures, including patient management, data collection, storage, analysis, and reporting, were conducted in accordance with institutional policies on data protection and confidentiality, including the European Union General Data Protection Regulation (EU GDPR). Written informed consent was obtained from all participants prior to enrollment.

### 2.5. Statistical Analysis

Continuous variables were assessed for normality using the Shapiro–Wilk test and visual inspection of histograms. Normally distributed variables are presented as mean values with standard deviation (SD), whereas non-normally distributed variables are expressed as median values with interquartile range (IQR). Categorical variables are summarized as counts and percentages (%). Comparisons between the LTB and HTB groups were performed using the Wilcoxon rank sum test for continuous variables and Pearson’s χ^2^ test or Fisher’s exact test, as appropriate, for categorical variables.

The primary endpoint was the presence of HTB. Univariable logistic regression analyses were initially performed to assess the associations between baseline variables, including the TyG index, and HTB. Variables with *p* < 0.10 in univariable analyses, together with clinically relevant covariates, were considered candidates for multivariable logistic regression. A prespecified multivariable model including the TyG index, age, sex, diabetes mellitus, smoking status, chronic kidney disease, WBC count, and multivessel CAD was constructed. In addition, a stepwise selection procedure based on the Akaike information criterion (AIC) was applied to derive a more parsimonious multivariable model. Results are reported as odds ratios (ORs) and adjusted ORs (aORs) with corresponding 95% confidence intervals (CIs).

To explore the functional form of the association between the TyG index, treated as a continuous variable, and the probability of HTB, restricted cubic spline functions were fitted within the multivariable logistic regression framework. The incremental predictive value of the TyG index beyond established clinical and angiographic predictors was evaluated using receiver operating characteristic (ROC) curve analysis. Specifically, a baseline clinical model was defined a priori based on clinical relevance and prior evidence and included age, diabetes mellitus, chronic kidney disease, WBC count, and multivessel CAD. The TyG index was subsequently added to this baseline model, and discrimination was compared using the area under the ROC curve (AUC). Optimal cut-off values for the TyG index alone and for the TyG-augmented clinical model were determined using Youden’s index, with the corresponding sensitivity and specificity reported.

Secondary analyses examined the association of the TyG index with distal embolization and no-reflow using similar univariable and multivariable logistic regression models.

All statistical tests were two-sided, and a *p*-value < 0.05 was considered statistically significant. Statistical analyses were performed using R statistical software version 4.4.2 (R Foundation for Statistical Computing, Vienna, Austria).

## 3. Results

### 3.1. Study Population and Baseline Characteristics

A total of 309 STEMI patients undergoing pPCI were included in the analysis. Among them, 135 (43.7%) were classified as having HTB (mTIMI grade 4) and 174 (56.3%) as having LTB (mTIMI grades 1–3). Baseline characteristics by TB are presented in Table 1. Age, sex distribution and the prevalence of major cardiovascular risk factors (hypertension, diabetes mellitus, dyslipidemia, smoking, family history of CAD, chronic kidney disease and peripheral artery disease) were broadly comparable between the two groups. There was a numerically higher prevalence of known CAD (12% vs. 6.3%, *p* = 0.080) and active smoking (71% vs. 61%, *p* = 0.077) in the HTB group. Left ventricular ejection fraction did not differ significantly between groups (44 ± 10% vs. 45 ± 9%, *p* = 0.5). Several laboratory indices were significantly higher in patients with HTB. Compared with LTB, the HTB group had higher WBC counts (11.9 ± 4.4 vs. 10.9 ± 4.2 × 10^9^/L, *p* = 0.027), higher urea levels (43 ± 23 vs. 36 ± 19 mg/dL, *p* = 0.003) and higher creatinine (1.11 ± 0.60 vs. 0.95 ± 0.34 mg/dL, *p* = 0.008). Lipid parameters and high-sensitivity troponin T were similar between groups (total cholesterol 163 ± 44 vs. 167 ± 45 mg/dL, *p* = 0.5; LDL-cholesterol 97 ± 37 vs. 103 ± 38 mg/dL, *p* = 0.15; triglycerides 125 (94,159) vs. 118 (88,168), *p* = 0.4; hs-troponin T 2142 ± 3181 vs. 2145 ± 2761 ng/L, *p* = 0.2). Importantly, the TyG index was significantly higher in patients with HTB than in those with LTB (9.12 ± 0.62 vs. 8.92 ± 0.64, *p* = 0.004).

### 3.2. Angiographic and Procedural Characteristics

Procedural and angiographic findings are shown in Table 2. The distribution of culprit vessels and the extent of CAD were similar between HTB and LTB patients. However, HTB was associated with more adverse angiographic profiles. Distal embolization occurred in 50.0% of patients with HTB versus 9.2% of those with LTB (*p* < 0.001). Myocardial no-reflow was observed in 21.5% of the HTB group compared with 6.9% of the LTB group (*p* < 0.001). Final TIMI flow was also less favorable in the HTB group: final TIMI 3 flow was achieved in 71.1% versus 94.3% in LTB (*p* < 0.001). The HTB group also had worse intra-procedural TIMI flow, with the worst TIMI 0–1 observed in 86.0% vs. 56.3% of patients and TIMI 3 in only 4.4% vs. 9.2% (*p* < 0.001), and a more frequent presence of residual thrombus at the end of the procedure (21.0% vs. 1.7%, *p* < 0.001).

### 3.3. Association Between TyG Index and High Thrombus Burden

In univariable logistic regression analyses, higher TyG index was significantly associated with the presence of HTB (*p* < 0.05) (Supplementary Table S1).

In the multivariable model adjusting for age, sex, diabetes mellitus, smoking status, chronic kidney disease, WBC count and prior CAD, the TyG index remained independently associated with HTB (aOR = 1.62; 95% CI: 1.09–2.44, *p* = 0.018) (Table 3, A). Smoking (aOR = 1.77; 95% CI: 1.05–3.04, *p* = 0.034) and known CAD (aOR = 2.40; 95% CI: 1.05–5.73, *p* = 0.041) were also independent predictors of HTB.

A stepwise multivariable model based on the Akaike information criterion yielded very similar results. In this model, the TyG index independently predicted HTB (aOR = 1.61; 95% CI: 1.11–2.37, *p* = 0.014), while smoking remained a significant predictor (aOR = 1.77; 95% CI: 1.06–3.02, *p* = 0.032) (Table 3, B). Age and WBC count showed borderline associations, and prior CAD trended toward significance (*p* = 0.054).

### 3.4. TyG Index and Secondary Angiographic Outcomes

**Figure 1:**
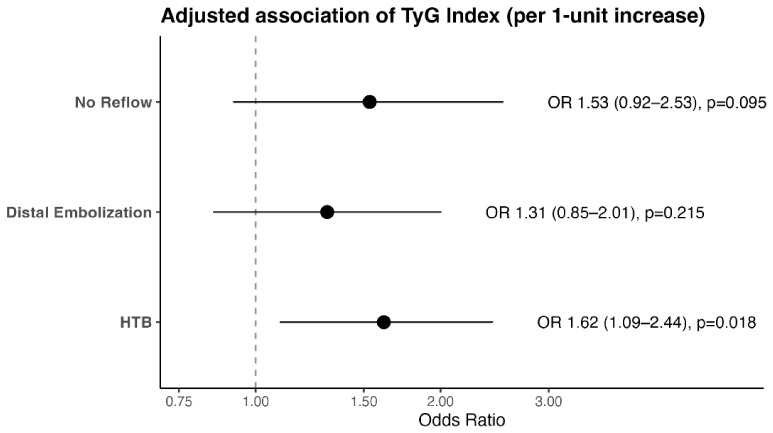
**Adjusted association of TyG index with angiographic outcomes.** Forest plot showing aORs and 95% CIs for the association between the TyG index (per 1-unit increase) and HTB, distal embolization, and no-reflow in patients with STEMI undergoing pPCI. The vertical dashed line represents the line of no effect (OR = 1.0). Abbreviations: aOR: adjusted odds ratio; CI: confidence interval; HTB: high thrombus burden; OR: odds ratio; pPCI: primary percutaneous coronary intervention; STEMI: ST-elevation myocardial infarction; TyG: triglyceride–glucose index.

The adjusted associations between the TyG index (per 1-unit increase) and the three angiographic endpoints, HTB, distal embolization and no-reflow, are summarized in the forest plot (Figure 1). In line with the primary analysis, higher TyG index was significantly associated with HTB (aOR = 1.62; 95% CI: 1.09–2.44, *p* = 0.018). For the secondary outcomes, higher TyG values were directionally associated with an increased risk of myocardial no-reflow (aOR = 1.53; 95% CI: 0.92–2.53, *p* = 0.095) and distal embolization (aOR = 1.31; 95% CI: 0.85–2.01, *p* = 0.215), although these did not reach statistical significance. Overall, the TyG index showed its strongest and most consistent association with angiographic TB, with weaker, non-significant trends toward complications of the microvasculature.

### 3.5. Predictive Performance of TyG Index for High Thrombus Burden

**Figure 2:**
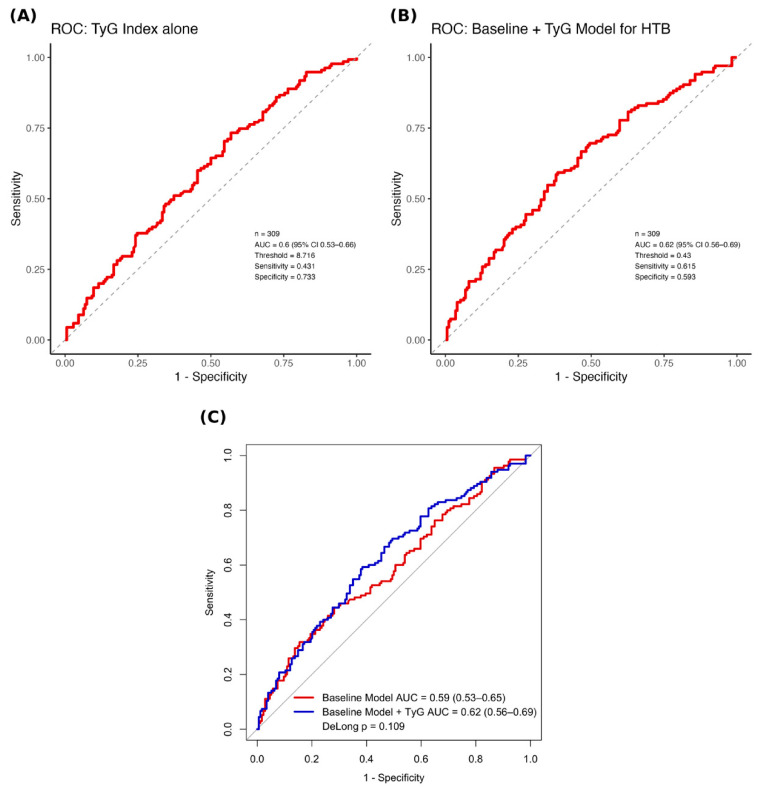
**Predictive performance of TyG index and baseline clinical model for high thrombus burden.** (**A**) ROC curve of the TyG index alone for identifying HTB in patients with STEMI undergoing pPCI. The area under the curve (AUC) is 0.60 (95% CI: 0.53–0.66). (**B**) ROC curve of the baseline clinical model plus the TyG index for identifying HTB. The AUC is 0.62 (95% CI: 0.56–0.69). (**C**) Comparison of receiver operating characteristic (ROC) curves between the baseline clinical model (red) and the baseline model augmented with the TyG index (blue). Abbreviations: AUC: area under the curve; CAD: coronary artery disease; CI: confidence interval; CKD: chronic kidney disease; HTB: high thrombus burden; pPCI: primary percutaneous coronary intervention; ROC: receiver operating characteristic; STEMI: ST-elevation myocardial infarction; TyG: triglyceride–glucose index; WBC: white blood cell.

In ROC analysis, the TyG index alone provided modest discrimination for HTB, with an area under the curve (AUC) of 0.60 (95% CI: 0.53–0.66) (Figure 2A). The optimal TyG cut-off identified using Youden’s index was 8.72, yielding a sensitivity of 43.1% and a specificity of 73.3% for identifying patients with HTB (Supplementary Figure S1A).

A baseline clinical model including conventional predictors of HTB including age, diabetes mellitus, chronic kidney disease, WBC, and multivessel coronary artery disease showed fair discrimination for HTB. When the TyG index was added to this model, the AUC increased numerically from 0.59 (95% CI: 0.53–0.65) to 0.62 (95% CI: 0.56–0.69), although this difference did not reach statistical significance by DeLong’s test (*p* = 0.109). (Figure 2B,C). Using the predicted probabilities from the combined model, the optimal probability threshold (Youden’s index) was 0.43, which corresponded to a sensitivity of 61.5% and a specificity of 59.3% for detecting HTB (Supplementary Figure S1B).

Restricted cubic spline analysis of the multivariable model confirmed a statistically significant association between the TyG index and the probability of HTB (overall *p* = 0.04), with no evidence of non-linearity (*p* for non-linearity = 0.59), supporting an approximately linear relationship across the observed TyG range (Supplementary Figure S2).

## 4. Discussion

### 4.1. Principal Findings

In this prospective STEMI cohort treated with pPCI, a higher admission TyG index was associated with a higher angiographic TB. The association remained independent after adjustment for clinical covariates in both a prespecified multivariable model and a parsimonious AIC-selected model. In addition, TyG showed an approximately linear, monotonic increase in the adjusted probability of HTB, supporting a continuous relationship rather than a threshold effect. TyG showed only modest standalone discrimination for HTB, and adding TyG to a baseline clinical model yielded only a small numerical improvement in AUC, suggesting a potential adjunctive predictive value. Finally, higher TyG values trended toward increased odds of distal embolization and no-reflow.

### 4.2. Pathophysiological Considerations

Several interrelated mechanisms may underlie the observed association between TyG and HTB. IR and metabolic syndrome are known to promote a systemic pro-oxidant and pro-inflammatory state and impair endothelial function, collectively shifting the hemostatic balance toward thrombosis [25]. In insulin-responsive tissues, insulin signaling normally supports vascular homeostasis by exerting mild anti-aggregatory effects through endothelial nitric oxide (NO) and cyclic nucleotide signaling and by promoting vasodilation [26,27,28]. In insulin-resistant states, this protective signaling is attenuated, favoring endothelial dysfunction, vasoconstrictor predominance, and platelet activation.

In parallel, IR is associated with an antifibrinolytic profile, including higher circulating levels of mediators such as plasminogen activator inhibitor-1 (PAI-1) and fibrinogen, which can facilitate thrombus persistence [29]. IR is also closely linked to atherogenic dyslipidemia further amplifying these processes [30,31]. Importantly, these links are supported by clinical evidence: higher TyG values have been associated with increased coagulation markers in patients with recent ACS [32]. Taken together, IR appears to synergistically prime the coronary vasculature for thrombosis but also weaken physiological restraints on thrombus propagation and microvascular perfusion.

### 4.3. Comparison with the Prior Literature

The current literature remains heterogeneous in endpoints and adjustment strategies, with most studies emphasizing clinical rather than angiographic outcomes. In a recent study of STEMI patients undergoing pPCI, Köktürk et al. reported that TyG independently predicted HTB (aOR = 1.47; 95% CI: 1.04–2.08, *p* = 0.02) [18]. Bilgin et al. similarly linked higher TyG with greater TB and a worse clinical course; in their dataset, high TyG was associated with increased short- and long-term mortality (HR = 2.5; 95% CI: 1.5–4.1, *p* = 0.01 and HR = 2.0; 95% CI: 1.3–3.2, *p* < 0.02, respectively) [2].

Beyond TB, prior work has also associated higher TyG values with PCI-related microvascular complications, particularly angiographic no-reflow. In STEMI patients with metabolic syndrome undergoing pPCI, Qu and Guan found that TyG ≥8.1 was the strongest independent predictor of no-reflow (OR = 9.591; 95% CI: 4.469–20.587, *p* < 0.001), with strong discrimination (AUC 0.869; sensitivity 0.791; specificity 0.891) [15]. In a separate STEMI cohort restricted to type 2 diabetes, Ma et al. likewise reported an independent association between TyG and no-reflow (OR = 3.23; 95% CI: 2.15–4.86, *p* < 0.001), with AUC 0.710 (cut-off 8.99; sensitivity 0.712; specificity 0.665) [33]. In contrast, although distal embolization is mechanistically linked to TB, the TyG–STEMI literature has largely focused on no-reflow, and, to our knowledge, angiographically defined distal embolization has not been evaluated as a standalone endpoint, making its inclusion in our analysis comparatively underexplored.

These angiographic observations may also help explain the broader prognostic signal consistently associated with TyG in ACS populations. A recent meta-analysis including over 20,000 patients showed that those in the highest TyG category had a significantly higher risk of MACCEs and all-cause mortality [34]. Complementary work has also linked higher TyG with post-PCI mortality in AMI patients and with composite endpoints when combined with other simple markers such as the neutrophil-to-lymphocyte ratio (NLR) or body mass index (TyG-BMI) [35,36].

More broadly, the association between glucometabolic dysregulation and adverse periprocedural angiographic outcomes extends beyond TyG. A recent multicenter prospective registry in NSTEMI patients undergoing PCI (*n* = 1005) demonstrated that the stress hyperglycemia ratio (SHR) was independently associated with type 4a myocardial infarction (aOR = 2.73; 95% CI: 1.70–4.42; *p* < 0.001) and with impaired final TIMI flow, suggesting that acute metabolic perturbations at the time of PCI may amplify thrombotic and microvascular injury irrespective of the specific index used [37].

### 4.4. Study Limitations

Our study has certain limitations that need to be acknowledged. First, this was a single-center, prospective observational study; therefore, residual confounding cannot be excluded and causal inference is limited. Second, although widely used in STEMI research, angiography provides only a qualitative estimate of thrombus and cannot characterize thrombus volume and composition with the granularity offered by intracoronary imaging [38,39]. Third, although TyG was independently associated with HTB, its standalone discrimination was modest (AUC~0.60), and the incremental gain beyond a baseline clinical model was small, indicating that TyG should not be interpreted as a definitive classifier of HTB in isolation. Fourth, symptoms to balloon or door-to-balloon time were not available for modeling; although all patients were treated within 6 h of symptom onset by protocol, variation in total ischemic time may still influence thrombus evolution and microvascular injury.

### 4.5. Clinical Implications and Future Directions

Because it is derived from routine laboratory tests, the TyG index can be calculated easily and at negligible cost, making it a practical adjunct for early risk stratification. However, given its modest discriminative performance when used alone, it should be interpreted as complementary to established clinical risk markers. Future research should focus on externally validating TyG-based risk enrichment for HTB and microvascular complications in larger, multicenter STEMI cohorts. Prospective studies could also examine whether TyG-guided intensification of antithrombotic or metabolic therapy translates into measurable reductions in early and/or late clinical outcomes.

## 5. Conclusions

In this prospective cohort of STEMI patients undergoing pPCI, a higher admission TyG index was independently associated with increased angiographic TB, with an approximately linear rise in the probability of HTB across the TyG spectrum. TyG provided only modest numerical incremental discrimination for HTB beyond conventional clinical variables and showed consistent, although non-significant, trends toward distal embolization and no-reflow.

Given that TyG is inexpensive, routinely available, and easy to calculate, it may serve as a useful adjunct for early risk enrichment in STEMI, complementing established clinical markers.

## Figures and Tables

**Table 1:** **Baseline characteristics by thrombus burden**.

Characteristic	Overall N = 309 ^1^	HTB N = 135 ^1^	LTB N = 174 ^1^	*p*-Value ^2^
Age, years				0.2
N	309	135	174	
Mean (SD)	60 (12)	61 (12)	60 (12)	
Gender				0.3
Female	24/309 (7.8%)	13/135 (9.6%)	11/174 (6.3%)	
Male	285/309 (92%)	122/135 (90%)	163/174 (94%)	
Hypertension	120/309 (39%)	52/135 (39%)	68/174 (39%)	>0.9
Diabetes mellitus	57/309 (18%)	29/135 (21%)	28/174 (16%)	0.2
Dyslipidemia	73/309 (24%)	31/135 (23%)	42/174 (24%)	0.8
Smoking	203/309 (66%)	96/135 (71%)	107/174 (61%)	0.077
Previous stroke or TIA	9/309 (2.9%)	2/135 (1.5%)	7/174 (4.0%)	0.3
Family history	73/309 (24%)	31/135 (23%)	42/174 (24%)	0.8
CKD	8/309 (2.6%)	2/135 (1.5%)	6/174 (3.4%)	0.5
PAD	8/309 (2.6%)	4/135 (3.0%)	4/174 (2.3%)	0.7
CAD	27/307 (8.8%)	16/133 (12%)	11/174 (6.3%)	0.080
BMI (kg/m^2^)				0.2
N	308	135	173	
Mean (SD)	28.9 (8.9)	29.8 (12.1)	28.2 (5.2)	
LVEF (%)				0.5
N	263	118	145	
Mean (SD)	44 (9)	44 (10)	45 (9)	
Hemoglobin (g/L)				0.2
N	306	134	172	
Mean (SD)	13.95 (2.55)	14.16 (3.40)	13.79 (1.60)	
WBC count (10^9^/L)				0.027
N	309	135	174	
Mean (SD)	11.3 (4.3)	11.9 (4.4)	10.9 (4.2)	
RBC count				0.2
N	306	134	172	
Mean (SD)	4.76 (0.87)	4.84 (1.12)	4.70 (0.61)	
Urea				0.003
N	308	135	173	
Mean (SD)	39 (21)	43 (23)	36 (19)	
Creatinine (mg/dL)				0.008
N	308	135	173	
Mean (SD)	1.02 (0.48)	1.11 (0.60)	0.95 (0.34)	
Total cholesterol (mg/dL)				0.5
N	286	123	163	
Mean (SD)	165 (45)	163 (44)	167 (45)	
LDL (mg/dL)				0.15
N	281	121	160	
Mean (SD)	100 (38)	97 (37)	103 (38)	
Triglyceride (mg/dL)				0.4
N	309	135	174	
Median (Q1, Q3)	122 (93,164)	125 (94,159)	118 (88,168)	
TyG index				0.004
N	309	135	174	
Meadian (Q1,Q3)	9.01 (8.58, 9.42)	9.13 (8.67, 9.48)	8.86 (8.48, 9.32)	
hsTroponin T (ng/L)				0.2
N	297	132	165	
Mean (SD)	2143 (2950)	2142 (3181)	2145 (2761)	

Abbreviations: BMI: body mass index; CAD: coronary artery disease; CKD: chronic kidney disease; HTB: high thrombus burden; hs-Troponin T: high-sensitivity cardiac troponin T; LTB: low thrombus burden; LDL: low-density lipoprotein cholesterol; LVEF: left ventricular ejection fraction; PAD: peripheral artery disease; RBC: red blood cell; TIA: transient ischemic attack; TyG: triglyceride–glucose index; WBC: white blood cell. ^1^ n/N (%). ^2^ Wilcoxon rank sum test; Pearson’s Chi-squared test; Fisher’s exact test.

**Table 2:** **Procedural and angiographic characteristics by thrombus burden**.

Characteristic	Overall N = 309 ^1^	HTB N = 135 ^1^	LTB N = 174 ^1^	*p*-Value ^2^
**Number of Diseased Arteries**				0.8
1	156/309 (50%)	71/135 (53%)	85/174 (49%)	
2	91/309 (29%)	37/135 (27%)	54/174 (31%)	
3	62/309 (20%)	27/135 (20%)	35/174 (20%)	
**Multivessel Disease (≥2)**	153/309 (50%)	64/135 (47%)	89/174 (51%)	0.6
LM	7/309 (2.3%)	5/135 (3.7%)	2/174 (1.1%)	0.3
LAD	204/309 (66%)	86/135 (64%)	118/174 (68%)	0.5
LCX	124/309 (40%)	52/135 (39%)	72/174 (41%)	0.7
RCA	196/309 (63%)	89/135 (66%)	107/174 (61%)	0.5
**Distal Embolization**	84/309 (27%)	68/135 (50%)	16/174 (9.2%)	<0.001
**Myocardial No Reflow**	41/309 (13%)	29/135 (21%)	12/174 (6.9%)	<0.001
**Final TIMI Flow 0–3**				<0.001
0	3/309 (1.0%)	2/135 (1.5%)	1/174 (0.6%)	
1	4/309 (1.3%)	4/135 (3.0%)	0/174 (0%)	
2	42/309 (14%)	33/135 (24%)	9/174 (5.2%)	
3	260/309 (84%)	96/135 (71%)	164/174 (94%)	
**Worst TIMI Flow 0–3**				<0.001
0	169/309 (55%)	99/135 (73%)	70/174 (40%)	
1	45/309 (15%)	18/135 (13%)	27/174 (16%)	
2	73/309 (24%)	12/135 (8.9%)	61/174 (35%)	
3	22/309 (7.1%)	6/135 (4.4%)	16/174 (9.2%)	
**Residual Thrombus**	32/309 (10%)	29/135 (21%)	3/174 (1.7%)	<0.001

Abbreviations: HTB: high thrombus burden; LAD: left anterior descending artery; LCX: left circumflex artery; LM: left main coronary artery; LTB: low thrombus burden; RCA: right coronary artery; TIMI: thrombolysis in myocardial infarction. ^1^ n/N (%). ^2^ Pearson’s Chi-squared test.

**Table 3:** **Multivariable regression model (A) and AIC-derived stepwise multivariable regression model (B) for thrombus burden**.

**Variable (A)**	**aOR**	**95% CI**	**β-Coefficient**	***p*-Value**
**TyG index**	1.62	(1.09–2.44)	0.48	0.018
Age	1.02	(1–1.04)	0.02	0.080
Male sex	0.53	(0.2–1.35)	−0.64	0.185
Diabetes mellitus	1.05	(0.55–2.01)	0.05	0.876
**Smoking**	1.77	(1.05–3.04)	0.57	0.034
CKD	0.26	(0.03–1.37)	−1.36	0.143
WBC count	1.04	(0.98–1.1)	0.04	0.167
**CAD**	2.40	(1.05–5.73)	0.88	0.041
Variable (B)	aOR	95% CI	β-coefficient	*p*-value
**TyG index**	1.61	(1.11–2.37)	0.47	0.014
CAD	2.28	(1–5.42)	0.83	0.054
**Smoking**	1.77	(1.06–3.02)	0.57	0.032
Age	1.02	(1–1.04)	0.02	0.091
WBC count	1.05	(0.99–1.11)	0.05	0.1

Abbreviations: aOR: adjusted odds ratio; CAD: coronary artery disease; CI: confidence interval; CKD: chronic kidney disease; TyG: triglyceride–glucose index; WBC: white blood cell.

## Data Availability

Study data will be available upon reasonable request from the corresponding study author (N.S.).

## Supplementary Materials

The following supporting information can be downloaded at https://www.mdpi.com/article/10.3390/jcm15124793/s1, Table S1: Univariable regression model for thrombus burden, Figure S1: Plots of sensitivity and specificity, Figure S2: Restricted cubic spline analysis of TyG index and high thrombus burden.

## Abbreviations

The following abbreviations are used in this manuscript: ACS: acute coronary syndrome; AIC: Akaike information criterion; AMI: acute myocardial infarction; AUC: area under the curve; aOR: adjusted odds ratio; BMI: body mass index; CABG: coronary artery bypass grafting; CAD: coronary artery disease; CI: confidence interval; CKD: chronic kidney disease; CRP: C-reactive protein; CVD: cardiovascular disease; eNOS: endothelial nitric oxide synthase; GDPR: General Data Protection Regulation; HDL: high-density lipoprotein; HR: hazard ratio; hs-Troponin T: high-sensitivity cardiac troponin T; HTB: high thrombus burden; IQR: interquartile range; IR: insulin resistance; LAD: left anterior descending artery; LCX: left circumflex artery; LDL: low-density lipoprotein; LM: left main coronary artery; LTB: low thrombus burden; MACCEs: major adverse cardiovascular and cerebrovascular events; MACEs: major adverse cardiovascular events; MI: myocardial infarction; mTIMI: modified thrombolysis in myocardial infarction; NLR: neutrophil-to-lymphocyte ratio; NSTE-ACS: non-ST-elevation acute coronary syndrome; NSTEMI: non-ST-elevation myocardial infarction; NO: nitric oxide; OCP: overall coagulation potential; OCT: optical coherence tomography; OR: odds ratio; PAI-1: plasminogen activator inhibitor-1; PAD: peripheral artery disease; PCI: percutaneous coronary intervention; pPCI: primary percutaneous coronary intervention; PI3K: phosphoinositide 3-kinase; RCA: right coronary artery; RBC: red blood cell; ROC: receiver operating characteristic; SD: standard deviation; STEMI: ST-elevation myocardial infarction; TB: thrombus burden; TIA: transient ischemic attack; TIMI: thrombolysis in myocardial infarction; TyG: triglyceride–glucose index; TyG-BMI: triglyceride–glucose body mass index; UA: unstable angina; WBC: white blood cell.
