# Supplementary material for: Association of Triglyceride–Glucose Index with Angiographic Thrombus Burden in Patients with ST-Elevation Myocardial Infarction: A Prospective Observational Study
Source: J Clin Med. 2026 Jun 20;15(12):4793. doi: 10.3390/jcm15124793 (PMC13301916; doi:10.3390/jcm15124793)
Supplement: Supplementary file 1 [file jcm-15-04793-s001.zip › Supplementary Table S1.pdf]

Supplementary Table S1. Univariable regression model for thrombus burden.

Variable	Odds Ratio	95% CI	β-coefficient	p-value
TyG Index	1.66	(1.16 – 2.43)	0.51	0.007
Age (years)	1.01	(0.99 – 1.03)	0.01	0.198
Male	0.63	(0.27 – 1.46)	-0.46	0.285
Hypertension	0.98	(0.61 – 1.55)	-0.02	0.920
Diabetes Mellitus	1.43	(0.8 – 2.55)	0.36	0.227
Dyslipidemia	0.94	(0.55 – 1.59)	-0.07	0.809
Smoking	1.54	(0.96 – 2.51)	0.43	0.078
Previous Stroke/TIA	0.36	(0.05 – 1.51)	-1.03	0.206
Family History of CAD	0.94	(0.55 – 1.59)	-0.07	0.809
Chronic Kidney Disease	0.42	(0.06 – 1.86)	-0.86	0.294
Peripheral Artery Disease	1.30	(0.3 – 5.58)	0.26	0.716
Known CAD	2.03	(0.92 – 4.64)	0.71	0.085
BMI (kg/m²)	1.03	(1 – 1.08)	0.03	0.195
LVEF (%)	0.99	(0.96 – 1.01)	-0.01	0.333
Hemoglobin	1.06	(0.97 – 1.2)	0.06	0.245
White Blood Cells	1.06	(1 – 1.12)	0.06	0.045
Red Blood Cells	1.24	(0.94 – 1.76)	0.22	0.177
Urea	1.02	(1.01 – 1.03)	0.02	0.005
Creatinine	2.26	(1.29 – 4.32)	0.82	0.008
Total Cholesterol	1.00	(0.99 – 1)	0.00	0.435
LDL	1.00	(0.99 – 1)	0.00	0.189
Triglycerides	1.00	(1 – 1)	0.00	0.649
hsTroponin T	1.00	(1 – 1)	0.00	0.993

*Abbreviations: BMI: body mass index; CAD: coronary artery disease; CI: confidence interval; HTB: high thrombus burden; LDL: low-density lipoprotein cholesterol; LVEF: left ventricular ejection fraction; OR: odds ratio; TIA: transient ischemic attack; TyG: triglyceride-glucose index.
