# Supplementary figures and images for: Association of Triglyceride–Glucose Index with Angiographic Thrombus Burden in Patients with ST-Elevation Myocardial Infarction: A Prospective Observational Study
Source: J Clin Med. 2026 Jun 20;15(12):4793. doi: 10.3390/jcm15124793 (PMC13301916; doi:10.3390/jcm15124793)

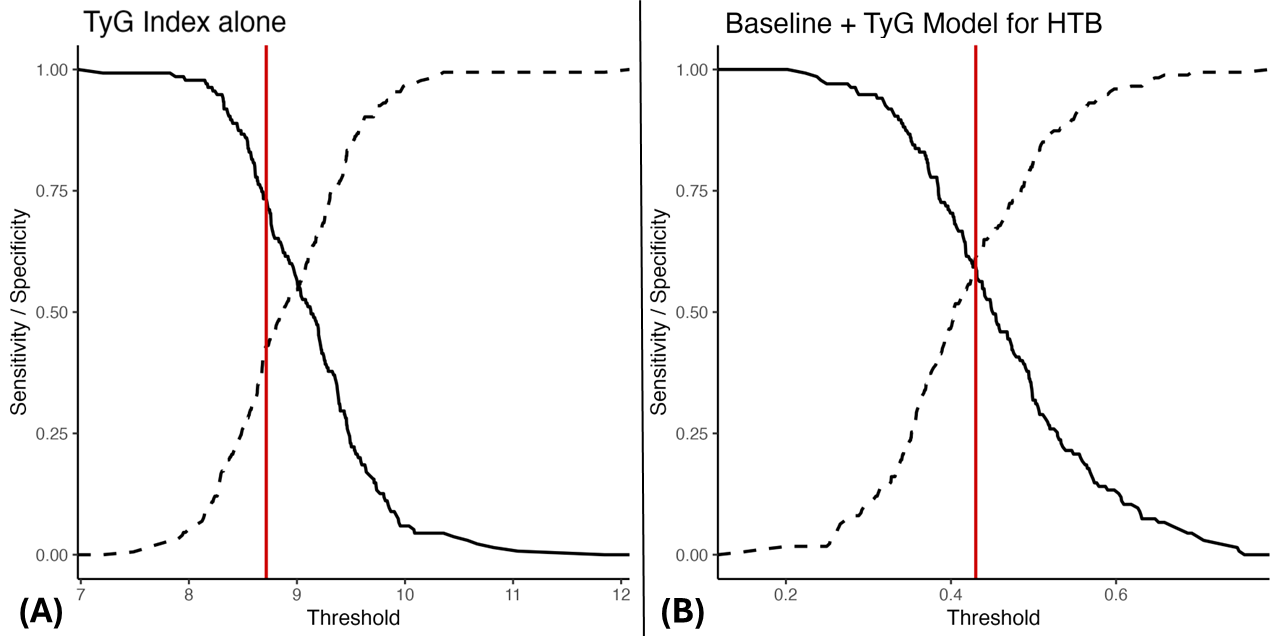

Supplement: Supplementary file 1 [file jcm-15-04793-s001.zip › Supplementary Figure S1. Plots of sensitivity and specificity..png]

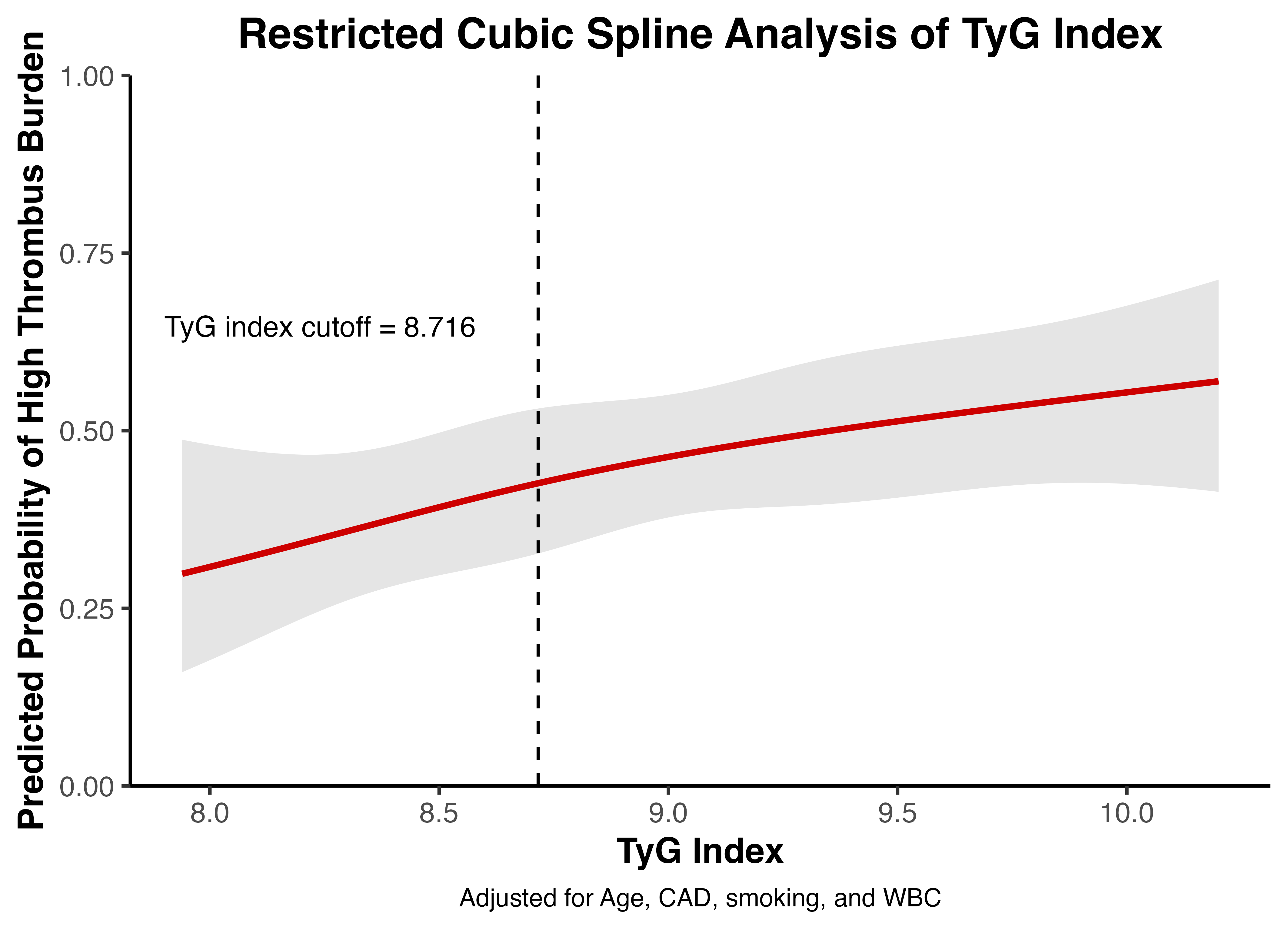

Supplement: Supplementary file 1 [file jcm-15-04793-s001.zip › Supplementary Figure S2. Restricted cubic spline analysis of TyG index and high thrombus burden..png]
